# Flexible 3D-Printable Materials for Application in Medical Research: A Comprehensive Comparison of Commercially Available Materials

**DOI:** 10.1089/3dp.2023.0179

**Published:** 2025-10-17

**Authors:** Maximilian Grab, Simone Jaud, Nikolaus Thierfelder, Christian Hagl, Bettina Wimmer, Markus Ahrens, Jan Stana, Petra Mela, Linda Grefen

**Affiliations:** ^1^ Department of Cardiac Surgery, LMU University Hospital, LMU Munich, Munich, Germany.; ^2^ Chair of Medical Materials and Implants, Department of Mechanical Engineering, TUM School of Engineering and Design, Munich Institute of Biomedical Engineering, Technical University of Munich, Munich, Germany.; ^3^ DZHK (German Centre for Cardiovascular Research), Partner Site Munich Heart Alliance, Munich, Germany.; ^4^ Division of Vascular Surgery, LMU University Hospital, LMU Munich, Munich Germany.

**Keywords:** 3D printing, medical, material testing, material characteristics, flexible, compliance

## Abstract

Additive manufacturing has become an important tool in modern medicine with numerous applications such as therapy planning, medical device prototyping and testing, and basic research. While the range of available materials continues to grow, the field of elastic printable materials for medical applications has not been fully explored. Therefore, this study focuses on the comparison of six commercially available flexible printing materials regarding their usability in the medical field. Surface of printed parts for all materials were analyzed directly after printing by scanning electron microscopy. The mechanical behavior was evaluated by uniaxial tensile tests, shore A hardness, and vascular compliance analysis. The degradation effects of the solvents water, glycerin, and ethanol were analyzed over a period of 7 days. The impact of different sterilization protocols (steam, plasma, ethylene oxide) was determined by tensile testing and surface imaging. Cytotoxicity was evaluated by a WST-1 proliferation assay with human endothelial cells. Microscopic imaging revealed a uniform surface with characteristic topologies depending on the printing technique. Different sterilization techniques revealed good results, however, pure silicone material TrueSil^®^ showed complete mechanical failure after plasma sterilization. All materials demonstrated high resistance to water and glycerin exposure with only slight weight changes. Ethanol exposure led to significant increases in wet weight with Agilus30 reaching a weight increase of 104.6%. All materials except Aceo showed a decrease in dry weight after ethanol exposure. Formlabs' Flexible 80A and Elastic 50A, as well as Agilus30, showed high cytotoxicity with no cell viability after 24 h. Aceo and TrueSil silicone materials revealed adequate cytocompatibility with viable cells even after 7 days. Static and dynamic vascular elasticity of all tested materials revealed linear elastic behavior. The tested materials offer unique material properties for different use cases, with the recapitulation of vascular elasticity still being a weak point of current printing technologies.

## Introduction

Additive manufacturing has become an important tool in industrial prototyping and even mass production. Over the last decade, the use of 3D-printing technology also steadily increased in the field of medicine, offering a wide range of possible applications,^1,2^ the most prominent of which is the planning of surgical and interventional procedures.^3–6^ Over the last years, 3D-printed patient-specific models have become standard planning tools in many medical disciplines ranging from maxillofacial surgery^1^ to pediatric cardiac surgery.^7^ 3D printing has also entered the area of surgical templates^8^ and even 3D-printed implants.^9^ In a laboratory setting, 3D printing has opened up new ways of designing and prototyping labortory equipment at a much lower budget (e.g., bioreactors), compared with traditional manufacturing techniques.^10–12^

Bridging the gap between clinical and basic science applications, 3D printing offers new ways of recreating anatomical structures for realistic *in vitro* tests, such as particle image velocimetry and 4D phase-contrast magnetic resonance imaging.^13,14^ The use of anatomical models allows a more precise development or selection of prostheses for individual patients. This has been especially true for the cardiovascular field, where patient–prosthesis mismatch is a possible complication.^15^

The success of all these applications relies in the availability of novel materials. The rise of 3D-printing applications in medicine made way for different commercial printing materials advertised as biocompatible or sterilizable. However, the commercially available materials for in-house printing are mostly limited to rigid properties with only few flexible materials. This is a constraint for many medical applications, resulting in expensive or time-consuming workarounds. Most notably, setups requiring flexible anatomical models rely on complex molds and dissolvable cores, significantly increasing the need for manual labor.^16,17^ The availability of flexible commercial materials is very limited and only very few are accessible for in-house printing. Nevertheless, flexible materials have found their way into the medical field, allowing for more accurate simulation of medical procedures^18–20^ and improved training models.^21^

Besides the advantages of accurate 3D-printed anatomical models, multiple studies describe limitations of current printing technologies such as cytotoxicity and a lack of tissue-mimicking properties.^22,23^ Furthermore, the comparable description of the properties of commercially available flexible materials is very limited.

Therefore, this work focuses on six of these flexible materials, comparing their properties for usage in the medical field, ranging from bioreactor parts to anatomical models for hemodynamic evaluation. Four of the tested materials are available for printing with popular 3D printers, accessible for many medical institutes. Two materials are pure 3D-printed silicones, which are only available through external manufacturing. The materials' properties will be compared concerning their cytotoxicity, sterilizability, resistance to solvents, basic mechanical parameters, and suitability to reproduce vascular compliance.

## Materials and Methods

The presented study didn't include patients or animals, therefore, ethical approval was waived by the Ethics Committee of the Ludwig Maximilians University, Munich.

### Material selection

In this study, six different flexible printing materials were analyzed (Table 1). To represent the most common available additive manufacturing techniques, two materials for Inkjet (*Agilus30 Clear FLX935*, Stratasys Ltd., Eden Prairie, MN, USA; *AR-G1L*, Keyence Corp., Osaka, Japan), two for stereolithography (SLA) (*Elastic 50A Resin*, Formlabs Inc., Somerville, MA, USA; *Flexible 80A Resin*, Formlabs Inc.), and two silicone printing technologies (*TrueSil 35A*, Spectroplast AG, Zurich, Switzerland; *ACEO Silicone GP Shore A50*, Wacker Chemie AG, Munich, Germany) were selected. AR-G1L was manufactured on an in-house printer (*Agilista-3200W*, Keyence Corp.). Similarly, Elastic 50A and Flexible 80A were printed using a Form 3 (Formlabs Inc.) available to the research group. All materials were postprocessed according to the manufacturer's instructions. Test specimens for Agilus30, TrueSil, and Aceo silicone were ordered from their respective manufacturers. All specimens were printed with 100% infill. All the conducted experiments are summarized in Figure 1.

**FIG. 1.**
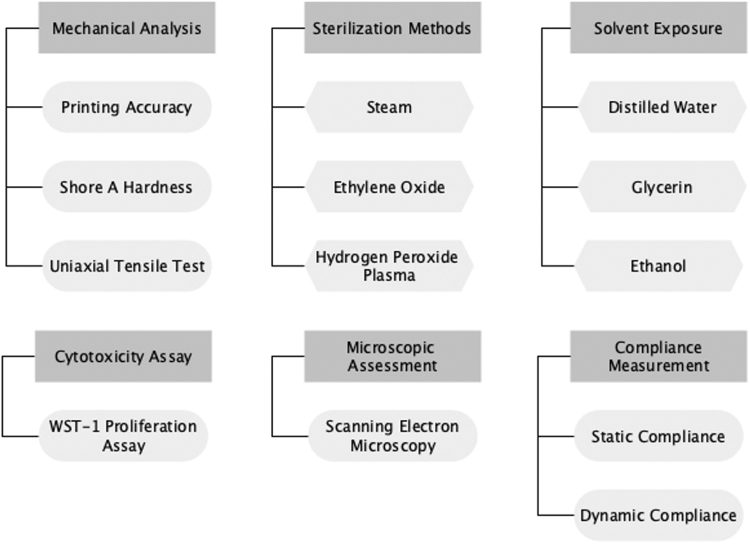
Overview of the applied methods.

**Table 1.** List of 3D-Printing Materials and Modalities Compared in This Study

	Material name	Manufacturer	Printing technology	Printer	Printing resolution (μm)
1	Agilus 30 Clear FLX935	Stratasys Ltd.	PolyJet	Stratasys Objet500 Connex 3	30
2	AR-G1L	Keyence Corp.	Inkjet	Keyence Agilista 3200W	30
3	Elastic 50A Resin	Formlabs Inc.	Stereolithography	Formlabs Form 3	100
4	Flexible 80A Resin	Formlabs Inc.	Stereolithography	Formlabs Form 3	50
5	Aceo Silicone GP Shore A50	Wacker Chemie AG	Silicone additive manufacturing	Specimen bought from the manufacturer	50
6	TrueSil A35	Spectroplast AG	Silicone additive manufacturing	Specimen bought from the manufacturer	100

### Mechanical analysis

Printing accuracy was assessed using a laser scanner. Briefly, WST-1 specimens (*n* = 10) were scanned (Autoscan Inspec, SHINING 3D Tech Co., Ltd., Hangzhou, China) for every material, and divergence in *x-*, *y*-, and *z*-axis was assessed by comparing the scan with the underlying stl-file. To assess the mechanical properties of the different materials, Shore A hardness of cylindrical samples (*n* = 5) with a diameter of 60 mm and thickness of 6 mm was measured using a durometer according to ISO 48-4 (HBA 100-0, Sauter GmbH, Balingen, Germany; Supplementary Fig. S1). Shore A hardness was measured after 3 and 15 s of constant indentation.

Furthermore, maximum tensile strength and elongation at break were determined by uniaxial tensile testing (zwickiLine Z2.5, ZwickRoell AG, Ulm, Germany). Dog-bone specimens (DIN 53504:2017 S3) were printed with three different thicknesses (1, 2, and 3 mm; *n* = 20 each) to assess the impact on the mechanical strength of the material.

### Sterilization methods

To determine the impact of different sterilization techniques on the flexible printing materials, dog-bone-shaped tensile testing specimens (DIN ISO 53504 S3) with a thickness of 2 mm (*n* = 20) were sterilized using the most commonly available sterilization techniques in a hospital setting: steam, ethylene oxide (ETO), and hydrogen peroxide plasma. Steam sterilization was conducted at 134°C and 3.1 bar pressure (Selec.PL9618-2GL, MMM GmbH, Planegg, Germany). Plasma sterilization using hydrogen peroxide was performed at 60°C for 75 min with a combination of ultraviolet (UV)-exposure and negative pressure (400 mtorr) (Sterrad 100S, ASP Global Manufacturing, Irvine, CA, USA). ETO sterilization was carried out at 45°C and 1.7 bar for 180 min, following a standard protocol. Unsterilized specimen with the same thickness served as controls.

### Solvent exposure

Material resistance to different solvents was determined following an adapted test protocol combining ISO 175:2011 and ISO 62:2008. As test specimens, 24 square plates (40 × 40 mm) with a thickness of 1 mm were printed for every material. Thereafter, samples were dried in an incubator at 50°C for 264 h to reduce residual humidity. The specimens were divided into four groups (each *n* = 6), including one group as native control. The samples of the treatment groups were put into separate glass bottles with 300 mL of the respective solvent. Six samples were covered with distilled water, six samples with glycerin (glycerin SOLVAGREEN^®^ ≥98%, Carl Roth GmbH & Co. KG, Karlsruhe, Germany), and six samples with ethanol (ethanol 99.8%, Carl Roth GmbH). For each sample, the weight *m_0_* was measured before submersion in the solvent using a precision scale (KERN 770, Kern & Sohn GmbH, Balingen, Germany).

Both untreated samples and submerged samples were stored at room temperature in a container to prevent additional UV exposure. Weight measurements were performed after 2, 4, 24, 48, 72, 96, and 168 h, respectively. After the last weight measurement, samples were transferred to an incubator at 50°C to detect possible weight loss in the dry material.

### Cytotoxicity assay

For the application of 3D-printed materials in medical research, their cytotoxicity is an important factor to be considered. Therefore, a WST-1 proliferation assay was performed for all materials. Following ISO 10993-12:2019, material specimens (*n* = 6 for each extraction interval and each cell line) were autoclaved and transferred to a sterile 8 mL tube (screw cap tube, 8 mL, Sarstedt AG & Co. KG, Nümbrecht, Germany) with 2.8 mL of endothelial cell culture medium (Endothelial Cell Growth Medium, Promocell GmbH, Heidelberg, Germany). Extraction was conducted for 24, 48, 72, and 168 h. After reaching the respective extraction time point, the medium was transferred to a 24-well plate, which had been seeded with human endothelial cells (50,000 cells/well) isolated from vena saphena magna pieces 24 h prior. This was repeated for a total of four cell lines.

After transferring the extract to the seeded wells, the well plates were incubated for an additional 24 h. Additional wells with native endothelial cell culture medium were seeded with the same cell density serving as control. Following the WST-1 assay instructions, the WST-1 assay reagent (Cell Proliferation Reagent WST-1, Roche Diagnostics GmbH, Mannheim, Germany) was incubated with the cells and finally transferred to a 96-well plate to measure absorbance at 450 nm (Multiskan EX, Thermo Fisher Scientific, Waltham, MA, USA).

### Microscopic assessment

To visualize the materials' surface structure, samples were visualized using scanning electron microscopy (SEM). All samples were dried in an incubator at 50°C and sputter-coated with gold for 180 s (SCD 50, Baltec GmbH, Schalksmühle, Germany). Subsequently, samples were visualized by SEM (Zeiss EVO LS 10, Carl Zeiss AG, Oberkochen, Germany) at different magnifications. Untreated samples served as controls.

### Static and dynamic compliance measurement

Since 3D-printed anatomical models are often used to represent flexible or elastic tissues (i.e., vascular models), the compliant behavior of the material is of utmost interest when choosing a printing material. For this reason, tubes with an inner diameter of 24 mm, a wall thickness of 3 mm, and a length of 200 mm were printed from all materials (*n* = 3), except TrueSil since the dimensions exceeded the printers' build plate size.

For static compliance measurements, the tubes were sealed and the pressure inside the vessels was increased in 20 mmHg increments using a 20 mL syringe. To determine their dynamic compliance, new tubes were inserted in a flow loop setup, consisting of a ventricular assist device (EXCOR VAD 60 mL, Berlin Heart GmbH, Berlin, Germany) as pulsatile pump, a laser micrometer (LS7070MR, Keyence Inc.), two pressure sensors (A-10, WIKA SE & Co.KG, Klingenberg, Germany), a reservoir, and a variable resistance (Supplementary Fig. S2). The assist device was actuated at a rate of 60 bpm, creating a systolic pressure of 120 mmHg in the vessel specimen, while dropping to a diastolic pressure of 80 mmHg. Dynamic compliance was measured for 5 min for every specimen. To calculate dynamic compliance, the following equation was used:

$$C_{dyn}=\frac{d_{sys}-d_{dias}}{d_{dias}}*\frac{1}{dp}*10^{4}\left[100\%∕mmHg\right]$$

where d_sys_ and d_dias_ are the measured systolic and diastolic diameters, respectively, and dp is the pressure difference between systole and diastole in mmHg.

### Statistical analysis

The statistical analysis was performed with GraphPad Prism 9.3.0 (GraphPad Software, San Diego, CA, USA). Normality was evaluated with the Kolmogorov–Smirnov test. Normally distributed variables are expressed as mean ± standard deviation. Groups were compared using one-way analysis of variance. A *p*-value ≤0.05 was considered statistically significant, while *p* ≤ 0.01 was considered highly significant and *p* ≤ 0.001 extremely significant.

## Results

### Print quality, microscopic and macroscopic assessment

Specimens ordered from Stratasys, Wacker, and Spectroplast offered high printing quality with no macroscopic differences. AR-G1L samples printed in-house also showed no divergence from the underlying .stl-files. On the contrary, both Formlabs materials proved to be difficult to print resulting in multiple misprints and deformed sample edges (Fig. 3A, B). Furthermore, the necessary attachment points for the support structures lead to a rougher surface. All printed samples appeared translucent, with AR-G1L (Fig. 2A.2) and TrueSil (Fig. 2A.6) being slightly opaque (Fig. 2A).

**FIG. 2.**
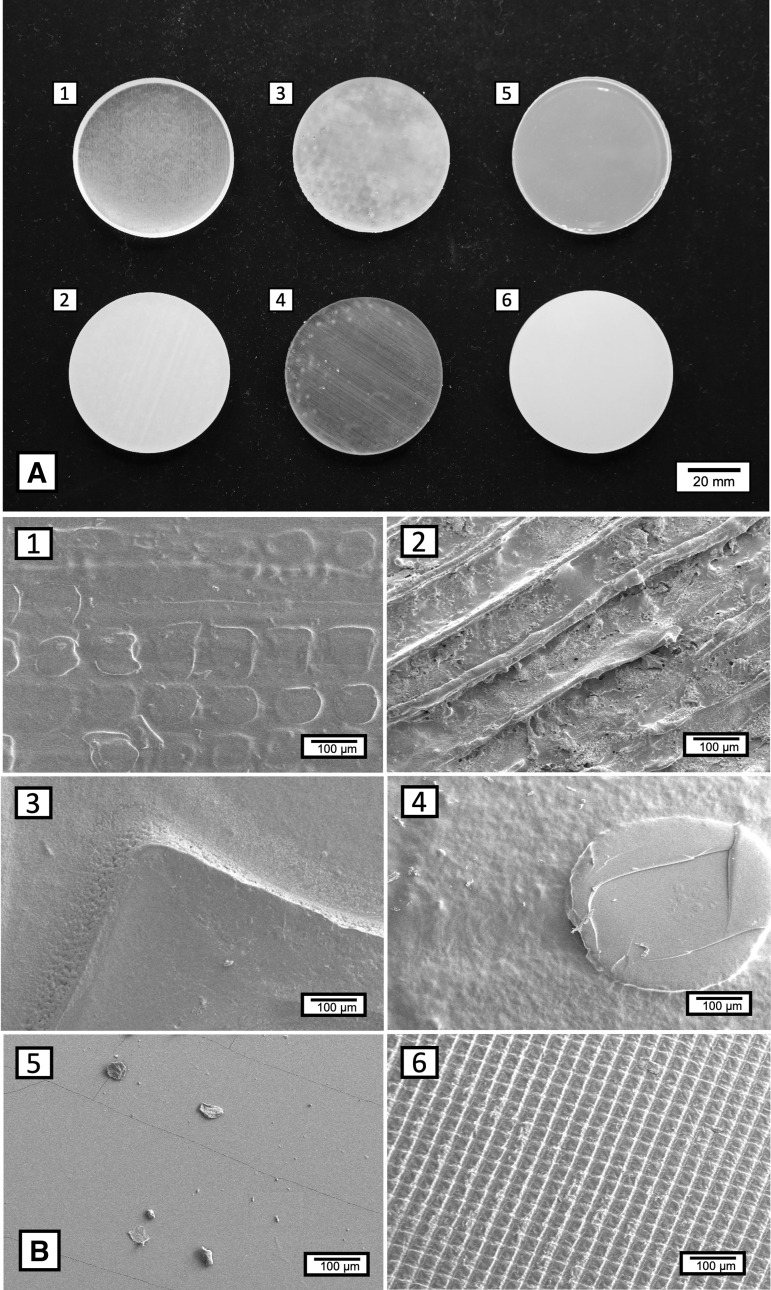
Macroscopic and microscopic evaluation of printed materials. **(A)** Color and opacity of materials. 1: Agilus30; 2: ARG1L; 3: Elastic 50A; 4: Flexible 80A; 5: Aceo; 6: TrueSil. **(B)** SEM imaging of native material samples. 1: Agilus30; 2: AR-G1L; 3: Elastic 50A; 4: Flexible 80A; 5: Aceo; 6: TrueSil. SEM, scanning electron microscopy.

**FIG. 3.**
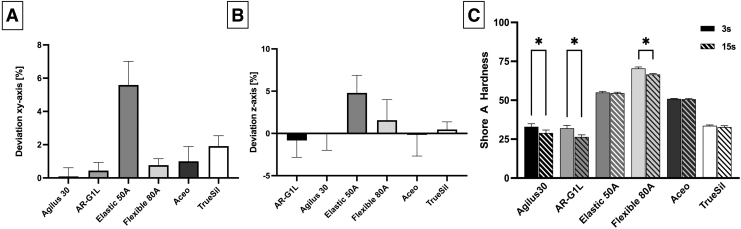
Printing accuracy and Shore A hardness. **(A)** Printing deviation in XY-direction. **(B)** Printing deviation in Z-direction. **(C)** Shore A hardness measured at 3- and 15-s indentation. *p < 0.05.

It was possible to print all the test specimens from all materials except for the compliance tube, which, unfortunately, could not be printed with Spectroplast TrueSil, since the required tube size exceeded the build-plate dimensions. SEM imaging of native samples revealed printing patterns characteristic for Inkjet technology (Fig. 2B.1, B.2) and SLA layering (Fig. 2B.3, B.4). In addition, support structure knobs were visible for the Flexible 80A material (Fig. 2B.4).

### Mechanical analysis

Measurement of Shore A hardness showed reliable and reproducible values for all materials (Fig. 3C). Agilus30 (nominal: 30 Shore A; 3 vs. 15 s: *p* < 0.001), AR-G1L (nominal: 35 Shore A; 3 vs. 15 s: *p* < 0.001), and Flexible 80A (nominal: 80 Shore A; 3 vs. 15 s: *p* < 0.001) showed progressing material indentation caused by the durometer, resulting in a significant reduction of Shore A hardness after 15 s of indentation compared with 3 s. For the remaining materials, hardness values showed no significant change caused by the prolonged indentation. Based on the manufacturer's data sheet values, all materials reached the specified hardness levels.

To assess the impact of printing resolution (layer thickness) on mechanical strength, dog-bone-shaped samples with a thickness of 1, 2, and 3 mm were printed and compared (Fig. 4). Agilus30 showed no significant difference in the tensile strength among the different printing thicknesses, however, with a statistically significant difference in the elongation at break between 1- and 2-mm samples. AR-G1L showed a lower maximum tensile strength for samples at a thickness of 1 mm. When compared with the 2- and 3-mm samples, statistical analysis revealed a significant difference.

**FIG. 4.**
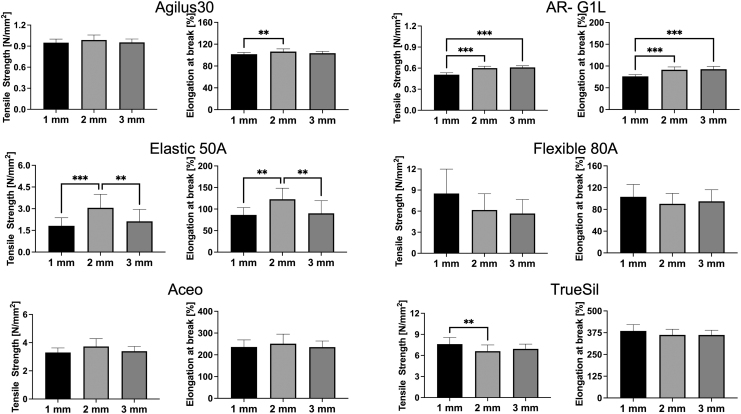
Mechanical properties for all selected materials as a function of sample thickness. Dog-bone-shaped test specimens were printed with a thickness of 1, 2, and 3 mm. **p < 0.01, ***p < 0.001.

This trend was also visible for the elongation at break, with a significant difference between 1-mm samples and the other specimens. Elastic 50A showed a very uneven behavior with the highest mechanical strength at 2-mm thickness and a significant reduction for both 1- and 3-mm samples. The 2-mm samples also performed best with regard to elongation at break, with a significant drop off for both 1- and 3-mm specimens. For Flexible 80A, samples showed a high variance within the groups and a decrease in mechanical strength with increasing thickness. Elongation at break on the contrary was comparable between the sample thicknesses. Aceo revealed very even behavior between the thicknesses with no significant change in mechanical strength or elongation at break. Maximum tensile strength of TrueSil showed a significantly higher value at 1-mm thickness compared with the 2-mm samples. The extensibility showed a more uniform behavior with no significant differences between the groups.

### Sterilization

Agilus30 revealed a significant increase in tensile strength for the plasma and ETO (control vs. plasma: *p* = 0.001; control vs. ETO: *p* = 0.004) groups (Fig. 5). Elongation at break for this material increased significantly for all sterilization groups compared with the control (control vs. steam: *p* = 0.002; control vs. plasma: *p* = 0.02; control vs. ETO: *p* < 0.001).

**FIG. 5.**
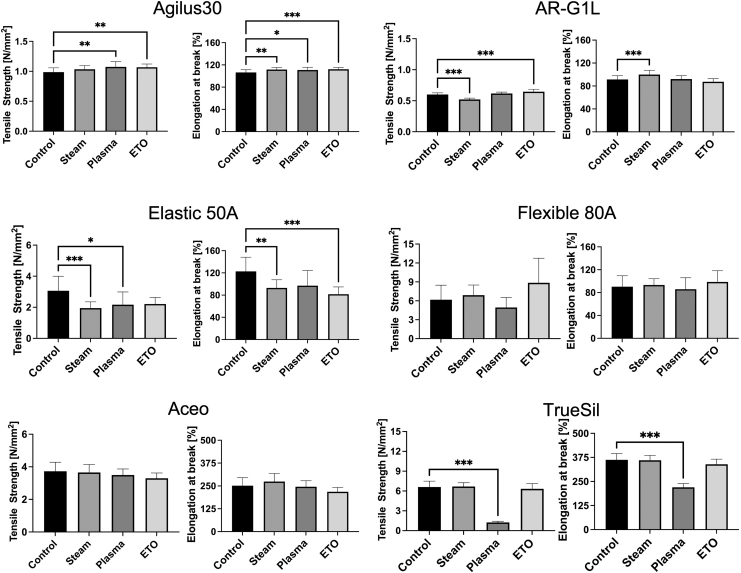
Impact of sterilization methods on the tensile strength and elongation at break of the selected materials. *p < 0.05, **p < 0.01, ***p < 0.001. ETO, ethylene oxide.

Mechanical assessment of the AR-G1L samples revealed a significant reduction of tensile strength for the steam-sterilized samples compared with the control (control vs. steam: *p* < 0.001).^24^ Contrary, elongation at break increased significantly for the steam-treated specimen (control vs. steam: *p* < 0.001).^24^ Plasma-treated samples of AR-G1L showed no change in tensile strength or elongation at break (control vs. plasma: *p* = 0.40), while ETO sterilization led to a significant increase in tensile strength (control vs. ETO: *p* < 0.001). In SEM imaging, steam-sterilized samples revealed large, fractured areas (Fig. 6B), an effect absent in native samples (Fig. 6A).

**FIG. 6.**
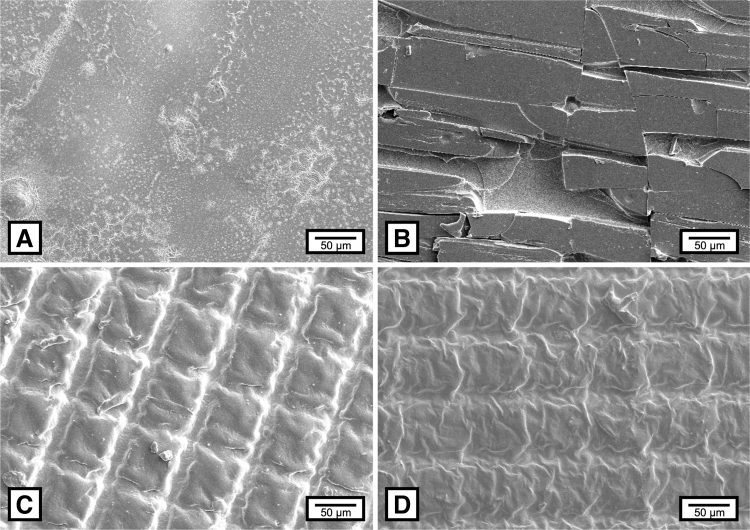
SEM imaging of sterilized samples. **(A)** Native AR-G1L sample. **(B)** AR-G1L sample after steam sterilization. **(C)** Native TrueSil sample. **(D)** TrueSil sample after plasma sterilization.

Elastic 50A showed a decrease in both tensile strength (control vs. steam: *p* < 0.001; control vs. plasma: *p* = 0.02; control vs. ETO: *p* = 0.06) and elongation at break (control vs. steam: *p* = 0.008; control vs. plasma: *p* = 0.06; control vs. ETO: *p* < 0.001) for all sterilization methods.

Flexible 80A displayed a mixed effect of the sterilization techniques with high variance between samples, causing elongation at break to stay on a comparable level to the control (control vs. steam: *p* > 0.99; control vs. plasma: *p* > 0.99; control vs. ETO: *p* = 0.88). Tensile strength increased for steam (control vs. steam: *p* = 0.97)- and ETO (control vs. ETO: *p* = 0.10)-treated samples, while tensile strength for the plasma samples decreased (control vs. plasma: *p* = 0.86). Compared with the other materials, Flexible 80A showed the highest standard deviation with regard to tensile testing.

Aceo silicone samples showed a uniform and unchanged behavior for both tensile strength (control vs. steam: *p* = 0.99; control vs. plasma: *p* = 0.62; control vs. ETO: *p* = 0.07) and elongation at break (control vs. steam: *p* = 0.57; control vs. plasma: *p* = 0.99; control vs. ETO: *p* = 0.12), with no significant difference caused by the sterilization methods.

For TrueSil silicone, tensile testing revealed a significant decrease in both tensile strength (control vs. plasma: *p* < 0.001) and elongation at break (control vs. plasma: *p* < 0.001) caused by plasma sterilization.^24^ During tensile testing, individual layers of the dog-bone specimens delaminated leading to total mechanical failure. For the other sterilization methods, TrueSil showed uniform behavior with no significant change in mechanical properties. Contrary to AR-G1L, TrueSil revealed only slight changes in the SEM images, showing a characteristic grid structure in both untreated and sterilized samples. After sterilization, the grid structure appeared more corrugated compared with native samples (Fig. 6C, D).

### Solvent exposure

Among the specimens exposed to water (Fig. 7A), Agilus30 and Elastic 50A showed swelling with a weight increase of 4.4 and 4.8%, respectively (Table 2). For all other materials, weight increased slightly (maximum 1.6%), but not significantly. After drying the samples, silicone-based materials retained an almost constant weight, while all other materials lost 0.5 (AR-G1L) to 1.2% (Agilus30) of the initial mass.

**FIG. 7.**
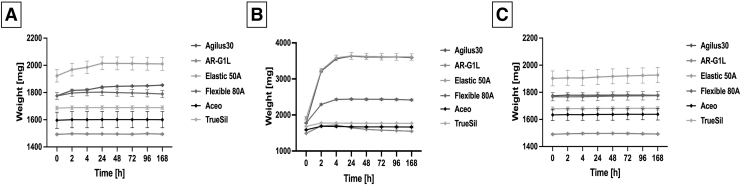
Solvent exposure of selected materials. **(A)** Weight change as a function of time of all materials submerged in water. **(B)** Weight change of all materials submerged in ethanol. **(C)** Weight change of all materials submerged in glycerin.

**Table 2.** Weight Change During and After Solvent Exposure

	Agilus 30		AR-G1L		Elastic 50A		Flexible 80A		Aceo		TrueSil	
Starting weight [mg]	1775.3 ± 2.9		1491.3 ± 4.2		1911.9 ± 47.7		1773.6 ± 24.5		1608.1 ± 45.1		1682.8 ± 11.7	

	Max. weight gain [%]	Weight after drying [%]	Max. weight gain [%]	Weight after drying [%]	Max. weight gain [%]	Weight after drying [%]	Max. weight gain [%]	Weight after drying [%]	Max. weight gain [%]	Weight after drying [%]	Max. weight gain [%]	Weight after drying [%]
Water	4.38 ± 0.16	−1.25 ± 0.03	0.44 ± 0.15	−0.49 ± 0.09	4.80 ± 0.06	−0.50 ± 0.02	1.57 ± 0.07	−1.22 ± 0.05	0.16 ± 0.03	−0.13 ± 0.05	0.22 ± 0.04	−0.07 ± 0.02
Ethanol	**104.63 ± 0.34**	−9.05 ± 0.07	15.40 ± 0.62	**−13.26 ± 0.26**	**90.60 ± 0.41**	−4.35 ± 0.14	37.24 ± 0.28	−4.44 ± 0.12	5.66 ± 0.12	−1.99 ± 0.04	5.57 ± 0.10	−1.57 ± 0.02
Glycerin	0.30 ± 0.08	−0.63 ± 0.03	0.53 ± 0.12	−0.43 ± 0.10	1.28 ± 0.08	0.53 ± 0.13	0.43 ± 0.04	−0.12 ± 0.04	0.12 ± 0.11	0.00 ± 0.04	0.19 ± 0.04	−0.08 ± 0.01

Bold numbers present noticeable weight change.

Max, maximum.

The most drastic weight changes were observed after exposure to ethanol (Fig. 7B). Except for the silicone-based materials, all tested materials showed large weight gain during exposure, with Agilus30 and Elastic 50A doubling their initial weight (104.6% and 90.6% weight gain).^24^ Silicone materials showed a slight increase in weight of 4.9% (Aceo) and 5.6% (TrueSil), followed by a slight weight loss after drying (Aceo: 3.0%; TrueSil: 1.6%).^24^ For the remaining materials, the drying process revealed a larger weight loss compared with the starting weight. Resin-based materials Elastic 50A and Flexible 80A showed a decrease of 4.4%, while acrylate-based materials Agilus30 and AR-G1L showed an increased weight loss of 9.1% and 13.3%.

When looking at the exposure to glycerin, all materials gained additional weight, ranging from 0.1% to 1.3% weight increase, during the exposure. After drying, all samples showed only small deviations from their initial weight (−0.6% to 0.5%; Fig. 7C).

### Cytotoxicity assay

Cytotoxic assessment revealed significant differences between the materials (Fig. 8A) Agilus30 and Elastic 50A, and Flexible 80A showed high cytotoxicity resulting in a reduction of cell viability by 100% already after 24 h of extraction. This, naturally, stayed constant for the 48- and 72-h mark, as well as the 7-day cultivation time point. For AR-G1L, the overall cell viability also decreased compared with the control group. After 24 h, photometric measurements revealed a reduced viability of 53.1 ± 11.7%. The cell viability stayed on a constant level for the remaining time points revealing an overall high cytotoxicity. After 24 h, silicone-based materials showed good adaptation of the endothelial cells to the extraction medium, resulting in a relative change of the cell proliferation rate of −1.3 ± 12.3% for Aceo and −12.9 ± 9.3% for TrueSil compared with the control.

**FIG. 8.**
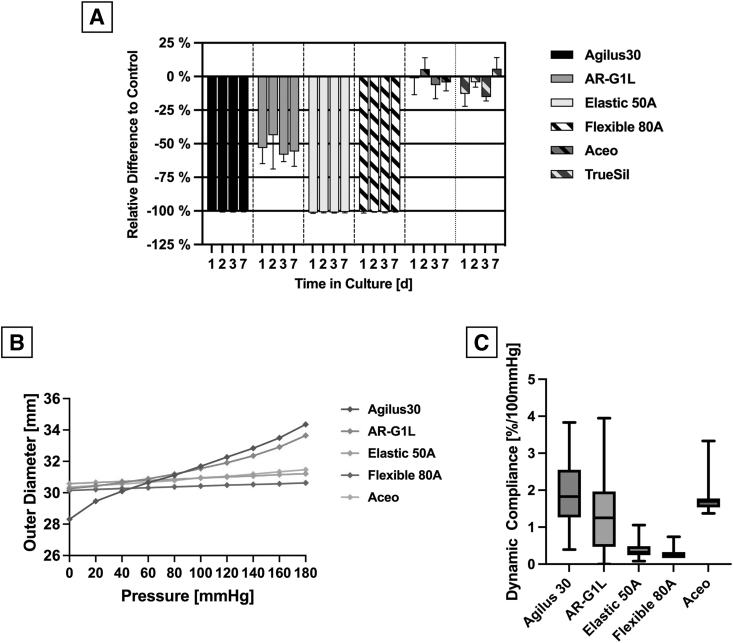
Cytotoxicity assay and compliance measurement. **(A)** WST-1 cell proliferation assay. Percentage of proliferation rate compared with a control group. **(B)** Static compliance of all materials measured by the increase in intraluminal diameter as a function of the applied pressure. **(C)** Dynamic compliance with a systolic pressure of 120 mmHg and a diastolic pressure of 80 mmHg.

This trend was observed for the 48-h (Aceo: 5.5 ± 8.4%; TrueSil: −4.3 ± 3.7%), 72-h (Aceo: −6.3 ± 10.2%; TrueSil: −15.3 ± 3.0%), and 7-day (Aceo: −4.5 ± 6.2%; TrueSil: 5.9 ± 8.2%) measurements as well, and thus, the two silicone-based printed materials were classified as noncytotoxic according to DIN EN ISO 10993-5:2009.

### Static and dynamic compliance measurement

Static compliance measurement of the printed tubes revealed the linear behavior of all materials across the pressure range from 0 to 180 mmHg. Agilus30 and AR-G1L showed a higher diameter increase due to the applied pressure compared with the other materials (Fig. 8B). Especially Agilus30 showed a significant increase in diameter from 28.3 to 34.4 mm (Δd = 21.5%; *p* < 0.001). Dynamic analysis of the samples' compliance revealed comparable compliance for Agilus30 (1.91 ± 0.86%/100 mmHg), AR-G1L (1.30 ± 0.92%/100 mmHg), and Aceo (1.67 ± 0.21%/100 mmHg; Fig. 8C).

## Discussion

Due to the compliant nature of many parts in the human system, flexible materials are a requirement in mock circulation setups, surgical models, as well as cell-based basic research. Mock flow loops often rely on off-the-shelf tube systems or silicone-molded phantoms.^16,17^ To create a higher degree of realism, anatomically correct phantoms are often incorporated in the flow loop setups. Creating molds of these complex anatomical structures requires sophisticated casting setups and adequate manpower and time, and therefore, simplifications are often required to create a reproducible phantom.^25–28^ In basic research, silicone is a popular material due to its favorable cytocompatibility, accessible price, and availability. Besides tubing, biocompatible flexible materials have also been used for gaskets, membranes, and cell chamber components.^29^ While these structures might be easier to create using molds due to their simpler geometries, undercuts and intricate structures are still difficult to form without soluble cores.

The introduction of additive manufacturing to the medical field offers a solution to these challenges present in a traditional mold-based model creation. In combination with segmentation software, it is possible to create realistic anatomical models that reproduce physiological structures.^13,30^ Furthermore, 3D printing has also found its way into the creation of bioreactor systems, allowing researchers to tailor their experimental setup more closely to their individual needs.^10,11,31,32^ Material researchers and companies have been reacting to this trend by developing novel materials to overcome the challenges present in medical research. Although most 3D-printing materials are stiff, a limited number of flexible materials are now available. The aim of our work was to compare these materials and provide this knowledge to the scientific community. The materials chosen for this work showed substantial differences in all properties tested, proving that this knowledge is important in the decision-making process for the individual application.

An overarching concern with the tested flexible printing materials is the very high cytotoxicity measured with a WST-1 assay. Both Formlabs materials Elastic 50A and Flexible 80A and the Stratasys material Agilus30 showed complete reduction of cell viability after 24 h, thus rendering these materials unsuitable for applications in cell-based environments. Cytotoxicity is a parameter that is not widely analyzed for flexible printing materials in the current literature. Kress et al. showed similar results when analyzing Formlabs Flexible 80A, showing a significant reduction in cell viability compared with the control.^22^ Due to their high cytotoxicity, the general application and work safety around these materials come into question. Toxic fume emission during the printing process and from the printed models has been a topic of recent research.^33,34^ Besides technical and laboratory personnel, this can also endanger patients, when the printed models are used as educational or planning models.

Since silicone models showed exceptional cytocompatibility, their implementation should be considered for cell-based printing projects, but also application in proximity to patients. While cytotoxicity is an important attribute in the ISO standard, there is a lot more testing required to deem a material biocompatible. Since this study had no specific biomedical application in mind, cytotoxicity was chosen as a comparative metric since it is required in every field of application mentioned in ISO 10993-12:2019.

Another property of importance when creating cytocompatible 3D-printed parts is the possibility to sterilize them. While most applied sterilization methods had only little impact on the mechanical properties, two specific combinations showed a drastic change. Especially the plasma sterilization of TrueSil led to total mechanical failure of the material. Most clinical plasma sterilization processes include UV exposure of the sterile goods. This UV exposure combined with increased temperature and negative pressure could have led to photodegradation of the material.^35,36^ Nevertheless, sterilization of flexible printing materials is possible, making the cytocompatible materials suitable for cell culture application.

In this study, both the silicone printing materials exceeded expectations and offered an alternative to cast silicone parts with regard to cytocompatibility, solvent resistance, and mechanical strength. Unfortunately, the tested materials are only available through production by the manufacturer, increasing the part cost and delivery time, compared with in-house printed options.

A property lacking for all tested materials is the adequate vascular compliance compared with the native porcine aortic tissue. Replicating the nonlinear elastic behavior of native tissue is a challenge multiple research groups are working on currently.^17,37,38^ Due to the nature of 3D printing, the anisotropic fiber orientation and thus the elastic restoring force of natural tissue are absent. This is a limiting factor, when using flexible models in mock circulation setups. Nevertheless, it allows for the creation of flexible aortic models in a much easier way compared with the classic molded models.

## Conclusions

The field of additive manufacturing is rapidly evolving and offers new possibilities for implementation in the medical field. The use of flexible printing materials in medicine is not yet widely described, but offers many fields of application, ranging from bioreactors to anatomical models. The tested materials offer unique material properties for many different use cases, with the reproduction of vascular compliance still being a weak point of the current printing technologies.
