# Supplementary material for: Flexible 3D-Printable Materials for Application in Medical Research: A Comprehensive Comparison of Commercially Available Materials
Source: 3D Print Addit Manuf. 2025 Oct 17;12(5):435–46. doi: 10.1089/3dp.2023.0179 (PMC12852178; doi:10.1089/3dp.2023.0179)
Supplement: Supplementary file 1 [file 3dp.2023.0179_suppl_figs1.docx]

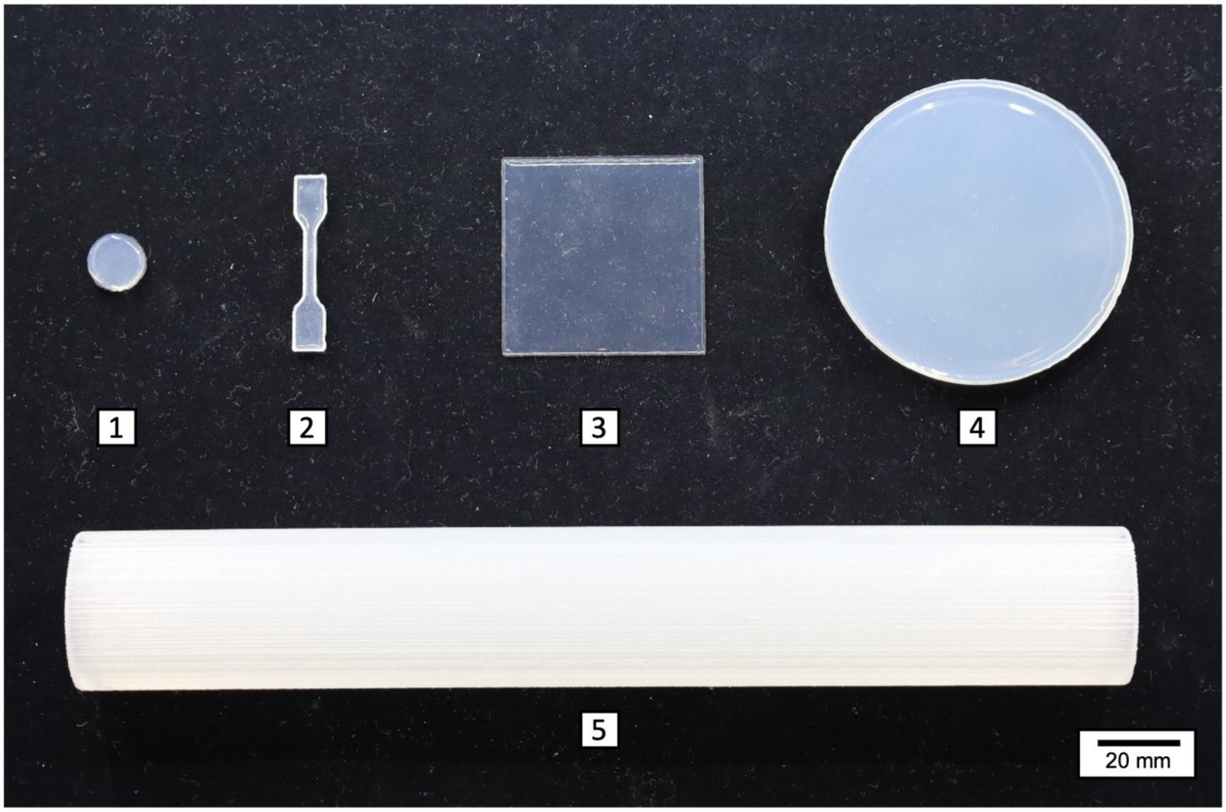
1
2
3
4
5
20 mm
