# Supplementary material for: Flexible 3D-Printable Materials for Application in Medical Research: A Comprehensive Comparison of Commercially Available Materials
Source: 3D Print Addit Manuf. 2025 Oct 17;12(5):435–46. doi: 10.1089/3dp.2023.0179 (PMC12852178; doi:10.1089/3dp.2023.0179)
Supplement: Supplementary file 2 [file 3dp.2023.0179_suppl_figs2.docx]

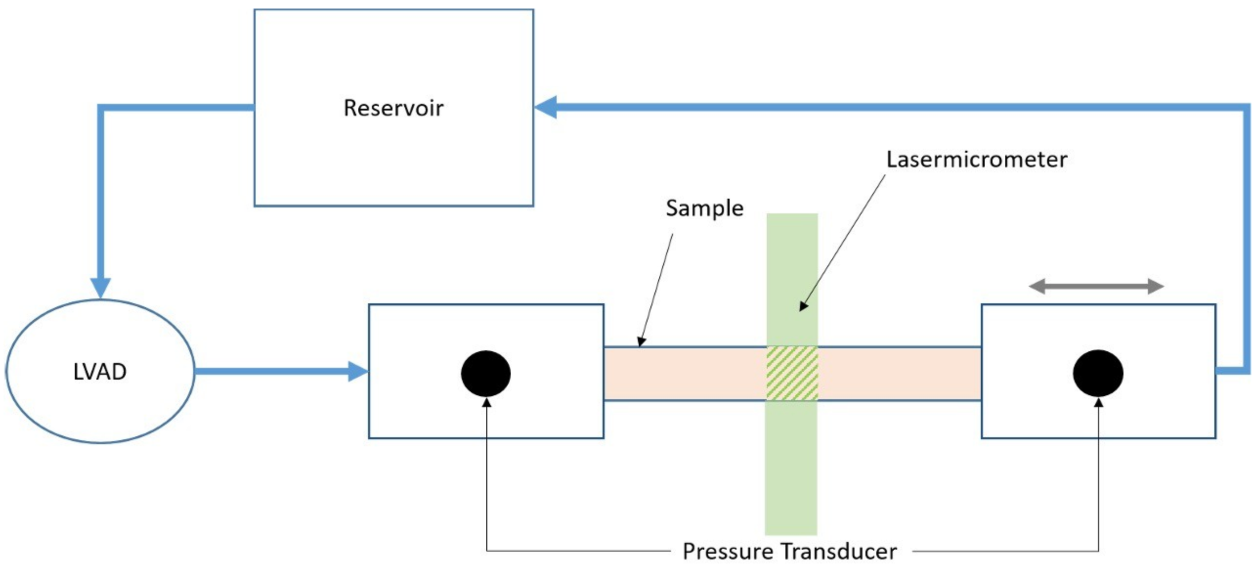
Reservoir
Lasermicrometer
Sample
LVAD
Pressure Transducer
